# Dynamic Detection of HbA1c Using a Silicon Nanowire Field Effect Tube Biosensor

**DOI:** 10.3390/bios12110916

**Published:** 2022-10-24

**Authors:** Hang Chen, Lijuan Deng, Jialin Sun, Hang Li, Xiaoping Zhu, Tong Wang, Yanfeng Jiang

**Affiliations:** 1 The First Affiliated Hospital of Nanchang University, Nanchang 330006, China; 2 Nanjing Medical University Affiliated Wuxi People’s Hospital, Wuxi 214043, China; 3 Internet of Things Institute, Jiangnan University, Wuxi 214122, China

**Keywords:** SiNW-FET, Debye shielding, HbA1c, diabetes, dialysis, dynamic detection

## Abstract

As an emerging diabetes diagnostic indicator and a dynamic change index, HbA1c can not only reflect the average blood glucose level over a period of time but can also well predict the incidence of related microvascular complications. It is important to develop a detection method that can dynamically characterize HbA1c. Silicon nanowire (SiNW) devices were mass-produced using top-down sputtering technology, and a microdialyzer was installed in a SiNW field effect tube biosensor detection system. Finally, the detection system was used to detect HbA1c levels quantitatively and dynamically in experimental rabbits. Various measurements showed that mass-produced SiNW devices have ideal dimensions, stable structures, and good performance. A series of microscopy results showed that the SiNW surface can be functionalized for intermolecular interactions. The addition of a dialysis device can effectively overcome Debye shielding, making the blood test similar to the pure standard test. Finally, the dynamic detection of HbA1c within 40 h was realized. SiNW biosensors are capable of the dynamic detection of biomolecules, and dynamic observation of the interaction between blood glucose and HbA1c provides new ideas for the diagnosis and treatment of patients with diabetes. Therefore, the SiNW biosensor can reflect the dynamic changes in HbA1c in a shorter time, which has a certain potential value in the clinical treatment of diabetes.

## 1. Introduction

In recent years, the incidence of diabetes has increased year after year. Data from the 2021 IDF Global Diabetes Map (10th Edition) show that the number of adults with diabetes worldwide in 2021 has reached 537 million and is expected to increase to 643 million in the next 10 years. Diabetes is a lifelong chronic metabolic disease, which is currently incurable. Its cause is relative and absolute deficiency of insulin, resulting in increased and uncontrolled blood sugar levels [1]. Current treatment modalities include long-term blood glucose monitoring and control; special diabetic dietary management; and the use of exogenous insulin, metformin, and other drugs [2,3]. Although at least one standard guideline for the diagnosis and treatment of diabetes is compiled every year in the world, the burden of diabetes on human health and the economy is still high [4]. Early diagnosis of diabetes and scientific and reasonable treatment and life management of the disease have become the most effective ways to reduce the harmful effects of diabetes. At present, the main methods of clinical diagnosis of diabetes include detection of fasting blood glucose levels, urine glucose levels, insulin levels, glycosylated hemoglobin levels, glucose tolerance, and islet cell antibodies [5]. In most cases, the diagnosis of diabetes requires a combination of multiple detection indicators, and sometimes it is necessary to repeat the detection of a certain indicator over a period of time [6,7,8]. This undoubtedly delays the early diagnosis of diabetes. In addition, long-term high blood sugar levels can cause damage to large blood vessels and microvessels and endanger the heart, brain, kidneys, peripheral nerves, eyes, and feet, causing a series of related organ and tissue complications [9,10]. Glycated hemoglobin has become one of the most important indicators for the diagnosis of diabetes in recent years [11]. Its level can not only be used as a direct criterion for the diagnosis of diabetes, but it can also predict the related microvascular complications of diabetes [12,13,14].

Glycated hemoglobin is the product of the slow, continuous, non-enzymatic combination of hemoglobin and glucose, and its level is related to the average blood sugar level. If the blood sugar is high for a long time, the test value of glycated hemoglobin will be higher than normal. The most representative one is HbA1c, which accounts for 70% of glycosylated hemoglobin, and it has good stability and is the best choice for clinical use [15]. Since HbA1c is a slow dynamic change indicator, it is different from the instantaneous and large changes in blood sugar level, and its level can better reflect the average blood sugar level over a period of time. Clinically, HbA1c is usually determined by affinity chromatography or high-performance liquid chromatography, and its normal value ranges from 20.22 mmol/mol to 42.08 mmol/mol. As a regular indicator of change, its regularity may provide more information on the progression of diabetes. However, there is currently no detection method to dynamically characterize its changes in clinical practice. If a detection method that can dynamically detect HbA1c is found, it will have great clinical significance for the diagnosis and treatment of diabetes [16].

Silicon nanowire field effect tube biosensors (SiNW-FET) have excellent sensitivity and specificity; they do not require labels in the biological detection process and can quickly reflect detection results in real time [17,18,19,20,21]. Among them, characteristics such as real-time detection and fast response also provide SiNW-FET with the potential ability to dynamically detect biomolecules [22,23]. The detection principle of SiNW-FET is as follows [24,25,26]: Due to the existence of protein isoelectric points (PIs), biological protein molecules behave as charged particles in a physiological solution environment (pH = 7.35–7.45), and charge ability is related to PI. When PI > 7.45, protein molecules release protons to become negatively charged particles, while, when PI < 7.35, protein molecules protonate to become negatively charged particles. Based on the principle of antigen–antibody specific recognition and binding, we used chemical covalent bonds to link the relevant antibodies of the target detection molecules on the SiNW surface. Therefore, during the detection process of the sensor, the nanowire can specifically recognize the target molecule and bind it to the nanowire. Therefore, charged target molecules pile up during the detection process and form an electric field around the nanowire. This electric field will affect the aggregation or dispersion of charge carriers inside the nanowire, thereby changing the conductivity of the semiconductor nanowire and causing the current to change. The concentration of the target molecule determines the strength of the surrounding electric field, indicating the degree of influence on the current and conductivity. Therefore, during the detection process, we reflect the concentration of the target detection molecule through the change in the conductivity of the nanowire and the output current. This external electric field effect is often weakened by the Debye shielding effect, which often occurs when semiconductor sensors are used for biological detection. It is similar to a doubly charged ion layer, which can cancel the effect of a certain strength of the electric field around the silicon nanowire. The Debye shielding effect is generally strong in solutions with high salt concentrations.

**Figure 1:**
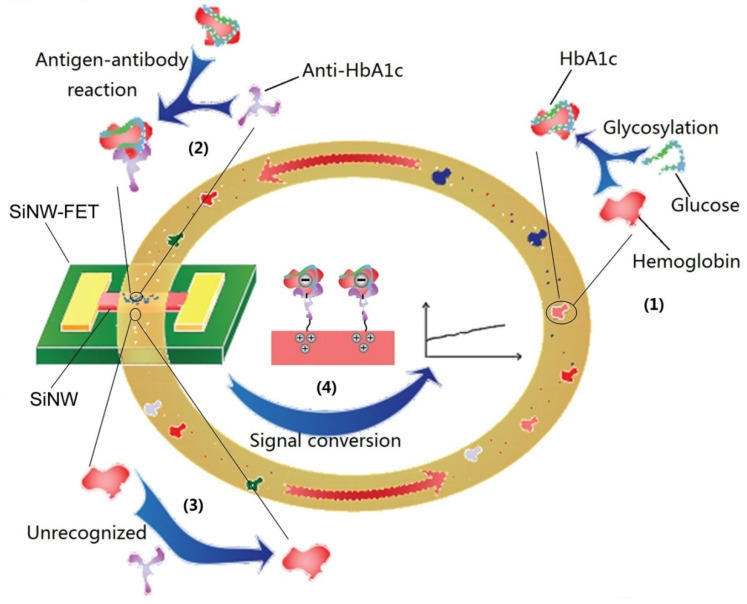
Schematic of the dynamic detection of HbA1c by SiNW-FET in circulating blood. (1) Nonenzymatic glycosylation of hemoglobin. (2) Antigen–antibody reaction of HbA1c, specific recognition, and binding. (3) HbA1c antibodies could not recognize hemoglobin. (4) The negative electric field effect makes the carriers in the silicon nanowires accumulate and enhances the output current.

The PI of HbA1c is about 6.8. In the detection solution, HbA1c molecules release protons into negatively charged particles. By modifying anti-HbA1c on the surface of silicon nanowires, the nanowires can specifically capture the HbA1c molecules in the detection solution and gather the HbA1c molecules on the surface of the nanowires, thereby forming an external electric field that can change the conductivity of the nanowires and then change the output current of the sensor. We used the dialysis method to purify the electrolyte in the detection solution and increase the Debye length, thereby weakening the Debye shielding effect and realizing the field effect of the external electric field on silicon nanowires. As chematic of the dynamic detection of HbA1c is shown in Figure 1. Over a period of time, high concentrations of blood sugar will slowly react with hemoglobin to generate HbA1c, thereby increasing the concentration of HbA1c. The increasing HbA1c also continuously enhances the external electric field effect on the nanowire surface, resulting in a gradual increase in the output current.

## 2. Materials and Methods

Silicon-on-insulator (SOI) wafers (p-type (100), ρ: 10–20 Ωcm) with a diameter of 6 inches, a Si layer thickness of 100 ± 10 nm, a silicon oxide layer thickness of 375 ± 5 nm, and a total thickness of 675 ± 15 μm were purchased from Nova Electronic Materials (Flower Mound, TX, USA). Silicon nanowire (SiNW) devices were fabricated by the Suzhou Institute of Nano-Tech and Nano-Bionics of the Chinese Academy of Sciences. HbA1c and anti-HbA1c were purchased from Abbexa (Cambridge, UK). Glycated albumin and anti-GA were purchased from Fitzgerald (Crossville, TN, USA). Bovine serum albumin (BSA), anti-BSA, and CD44 were purchased from Sigma-Aldrich (Saint Louis, MO, USA). Experimental rabbits and diabetic rabbit models were purchased from Ubanbio (Shanghai, China). The dialysis membrane was purchased from Fresenius Medical Care (Bad Homburg, Germany).

The SiNW device was fabricated via a top-down method. First, the Si layer was thinned to 30 nm by oxidation, and then nanowires with a height of 80 nm and a width of 300 nm were etched by photolithography and reactiveion etching (RIE). Next, a layer of silicon oxide with a thickness of 50 nm was plated on the nanowire using photolithography technology combined with inductively coupled plasma chemical vapor deposition (ICPCVD) technology. Next, the source, drain, and two gates were formed by using photolithography technology combined with Physical Vapor Deposition (PVD) technology, and all four electrodes were Ti (5 nm) + Au (100 nm) + Ti (5 nm). Then, the device was annealed (rate 10 °C/s, parameters: 200 °C × 30 s, 330 °C × 10 s, 100 °C × 60 s). Finally, a SiO_2_ (100 nm) + SiN_x_ (160 nm) passivation layer was formed by photolithography combined with ICPCVD technology, and Si nanowires with a length of 10 μm and four electrodes were exposed.

SiNW surface modification was performed to connect the antibody protein molecules with specific recognition and binding abilities on the surfaces of nanowires through linker chains, so that the sensor would have specific detection abilities. The linker chain was a covalent chain dominated by 3-aminopropyltriethoxysilane-glutaraldehyde (APTES-Glu). In the modification process, the surface of the device was first treated with an oxygen plasma cleaner for 5 min to produce OH- and then it was soaked in 2% (*v*/*v*) APTES ethanol solution for 45 min. After washing with ethanol, it was heated at 120 °C for 1 h. Then, it was soaked in 2.5% glutaraldehyde solution for 1 h and washed with deionized water. The prepared antibody solution of 100 μg/mL was dropped on the nanowire for 4 h, and finally it was washed with deionized water and stored at 4 °C for later use.

Taking the SiNW device as the main body, the synthesis of the detection system required reversible sealing of the micro-channel mold and addition of a microdialyzer. The micro-channel mold was made of polydimethylsiloxane (PDMS). The mold was solidified on the model and it underwent hydroxylation treatment and was finally reversibly sealed with the SiNW device. The entire detection system was connected by a thin silicone hose with an inner diameter of 0.5 mm. The microdialyzer was a miniature version of the clinical dialyzer, and the dialysis membrane had a pore size of 10,000 Daltons. It was connected between the specimen tube and the SiNW-FET.

The platform for the SiNW-FET detection experiments was a semiconductor analyzer (Agilent B1500A, Santa Clara, CA, USA), the source and drain voltages were set to 0.1 V, the top gate was −3 V (G_1_ = −3 V), and the back gate was 1 V (G_2_ = 1 V). SiNW-FET must meet the following conditions during detection; otherwise, the device must be judged unqualified: the average base current value of each device must be 0.4–0.6 μA, and the base current fluctuation range must not exceed 50 mA. And the range of ΔIds does not exceed 5% of the average ΔIds.

The detection experiment was divided into three rounds, and each round was repeated at least three times. The first round was the standard solution detection. The three groups of SiNWs modified with anti-GA, anti-HbA1c, and anti-BSA were named NW-1, NW-2, and NW-3, respectively, and then they were detected in the following sequence: (1) 0.02 × PBS, (2) glycated albumin (GA) standard 5 ng/mL, (3) HbA1c standard 5 ng/mL, (4) bovine serum albumin (BSA) standard 5 ng/mL, and (5) HbA1c standard 0.5 ng/mL. The detection time for each detection solution was 120 s (this time scale was chosen to show the stability of the results). Next, different concentrations of HbA1c standards were tested. The standard substance of antigen was prepared using 0.02 × PBS and pure antigen substance. The second round was the detection of HbA1c in rabbit blood. Rabbit blood with HbA1c concentrations of 34.43 mmol/mol, 55.19 mmol/mol, and 80.33 mmol/mol was detected. The third round of detection was dynamic detection. During dynamic detection, the rabbit was intubated under general anesthesia. Blood was collected from the ear vein with an indwelling needle; 100 μL of blood was collected every 15 min for detection, and the dynamic detection time was 40 h. The detection target was the three groups of experiments; the experimental rabbit 1 was a normal experimental rabbit, and normal feed solution (ter in die, 200 g/time) was injected from a gastric tube. The experimental rabbit 2 was a diabetic model rabbit, and the diet and frequency were the same as those for rabbit 1 (the feed ratio was 30% sucrose + 70% normal feed). The experimental rabbit 3 was a diabetic model rabbit, and its diet and frequency were the same as those for rabbit 1 (the feed ratio was 40% sucrose + 60% normal feed).

## 3. Results and Discussion

The experimental results for the SiNW-FET detection of HbA1c will be discussed in the following points. First, the basic performance of SiNW devices was verified, which was the basis for detection of SiNW-FETs. Second, it was necessary to clarify the premise of the detection and application of SiNW-FET, which was the ability to functionalize a modified antibody protein on the SiNW surface. Through the specific recognition and binding characteristics of antigen and antibody, the nanowire could recognize and capture the target detection molecule; thus, the modification and verification of the nanowire surface were also essential. The next problem to be solved was the problem faced by the semiconductor nanowire biosensors: how to overcome the Debye shielding effect during biological liquid detection? Finally, the real-time, high-specificity, high-sensitivity, and dynamic detection of HbA1c was verified by detection experiments.

**Figure 2:**
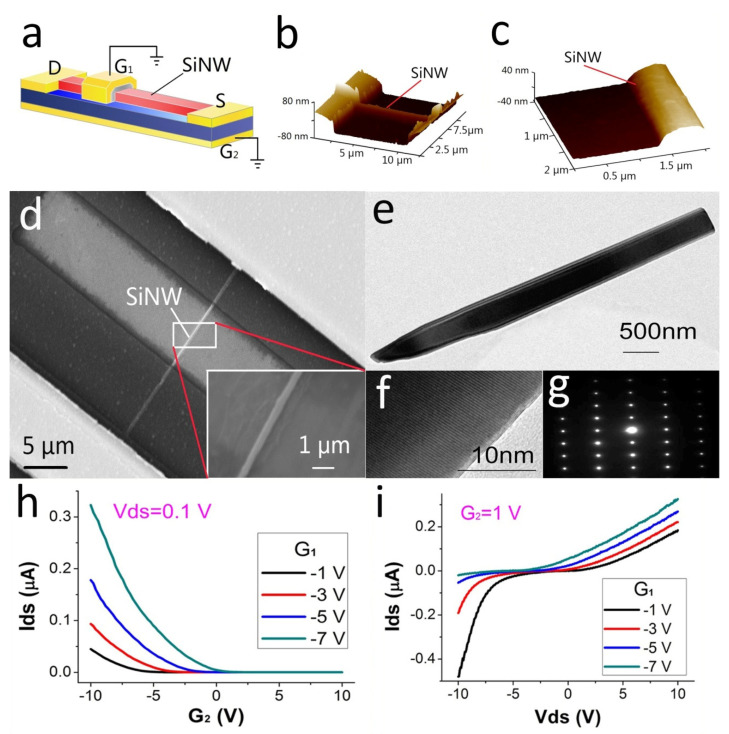
Fundamental properties of SiNW. (**a**) Schematic diagram of the electrode structure of the SiNW device. (**b**,**c**) AFM image of SiNW. (**d**) SEM image of SiNW. (**e**–**g**) TEM image of SiNW. (**h**) Transfer curves of SiNW device. (**i**) Output curve of the SiNW device.

Nanowire integrated circuit chips were fabricated via a top-down process. The raw materials of nanowire devices were p-type SOI wafers. Through photolithography technology, sputtering technology, and coating technology, double-gate SiNW devices could be mass-produced. The structure is shown in Figure 2a. The source (S) and drain (D) were connected by SiNW to form a loop. Two gates were arranged around the SiNW, which were the top gate (G_1_) and the back gate (G_2_). G_1_ and G_2_ formed a ring gate around the SiNW. To verify the reliability of our device’s mass-produced approach, we conducted structural and performance tests on the nanodevice. The height of the nanowire was measured via atomic force microscopy(AFM), as shown in Figure 2b–c. Its height was 80 nm. Next, we used scanning electron microscopy (SEM) to measure the nanodevices, as shown in Figure 2d. It can be seen that the integrity of the nanowires was good, and the line width was about 300 nm. We then used transmission electron microscopy (TEM) to characterize the nanowires, as shown in Figure 2e–g; it can be seen that the appearance and internal structure of the SiNW were stable. Finally, we tested the electrical properties of the SiNW device on a semiconductor analyzer. It can be seen that, in the transfer curve (Figure 2h), when the source and drain set the rated voltage (Vds), different gate voltages caused changes in the current (Ids) in the nanowire. The greater the gate voltage, the greater the influence on the current. Moreover, we examined the output curve of the device (Figure 2i), and we could see that Ids changed with Vds, and different gate voltages caused different degrees of current changes. Through the basic gate voltage performance test, it could be seen that the device had good gate voltage gating performance, which was the basis for the high sensitivity detection of SiNW-FET.

**Figure 3:**
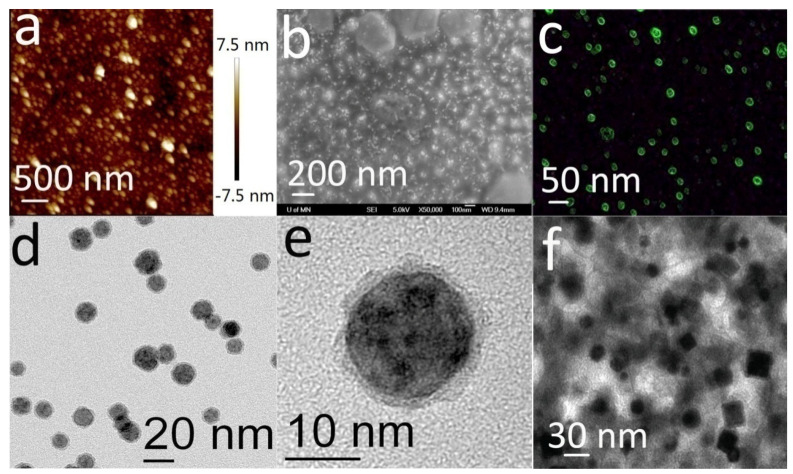
Surface modification and recognition of intermolecular recognition results of the SiNW device. (**a**) AFM image of the SiNW device modified with anti-HbA1c. (**b**) SEM image of the SiNW device modified with anti-HbA1c. (**c**) Fluorescence microscopy of CD44 protein modified through linker chains on SiNW. (**d**–**f**) TEM images of intermolecular recognition and binding on SiNW surfaces.

In order to realize the highly specific detection of biomolecules by SiNW-FET, we modified the SiNW surface. We modified the receptors that could specifically recognize and detect the target molecule (in most cases, the specific antibody of the target molecule was selected) as well as accumulate the target molecule around the nanowire through specific recognition and binding between biomolecules. In this experiment, SiNWs and antibody molecules were connected by the organic chain APTES-Glu. The microscopyresults of the molecular modification of the SiNW surface and the recognition of intermolecular recognition are shown in Figure 3. Figure 3a,b are AFM images and SEM images showing the anti-HbA1c modification on the surface of the SiNW device, respectively, which clearly show that the anti-HbA1c protein molecular particles attached to the surface of the SiNW device. Figure 3c is the fluorescence microscopyimage of the surface of the SiNW device after CD44 protein modification. The above results show that the SiNW device has good surface modification ability. Figure 3d−e are the TEM images of HbA1c standard solution detection after the modification of SiNW by anti-HbA1c protein, which clearly show the binding of antigen–antibody molecules. Figure 3f is the TEM image of SiNW modified by anti-HbA1c protein after blood detection, which shows the molecular binding. These results are the basis for demonstrating the specific detection of HbA1c by SiNW-FET.

**Figure 4:**
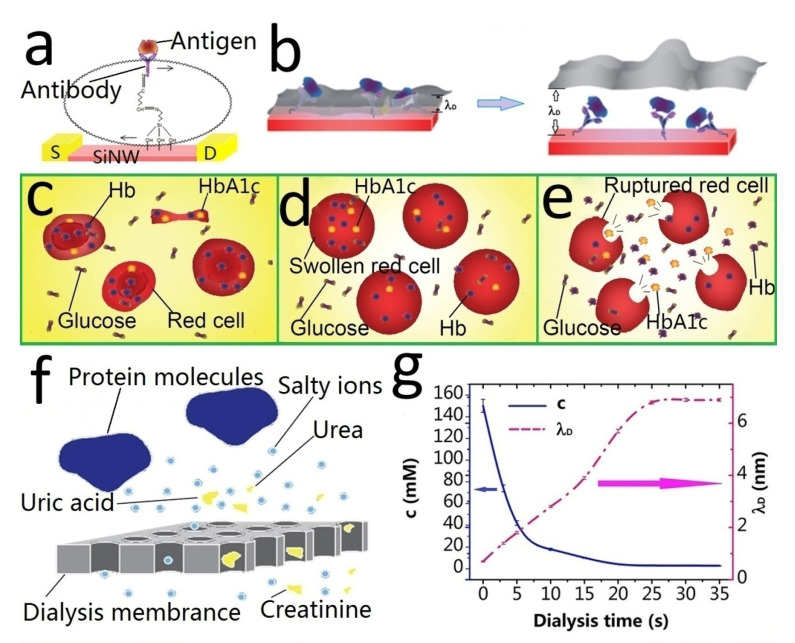
Principle of the Debye shielding effect and microdialyzer. (**a**) Schematic diagram of the electric field effect generated by antigen molecules during detection. (**b**) Schematic diagram of Debye length and Debye shielding effect. (**c**–**e**) Schematic diagram of the stepwise hemolysis process. (**f**) Principle of the dialysis membrane. (**g**) Ionic strength and Debye length of the solution during dialysis as a function of dialysis time.

As chematic diagram of the electric field effect generated by antigen molecules during the detection of biological solutions with SiNW-FET is shown in Figure 4a. However, as a semiconductor nanowire biosensor, the Debye shielding effect will be encountered in the detection of salt ion solutions, which can shield the sensor from the electric field effect caused by target molecules in physiological solutions, such as blood, serum, tissue fluid, and urine [27]. The principle of the Debye shielding effect is that the abundant salt ions in the biological solution form a doubly charged ion layer on the surface of the nanowires. This ionic layer can dissipate almost all of the electric field effects produced by target molecules when examining untreated physiological solutions [28]. The strength of the Debye shielding effect is related to the Debye length (λ_D_). The longer the λ_D_, the weaker the Debye shielding effect. In addition, λ_D_ is related to the following factors [29]: the ionic strength of the detection solution, the dielectric constant of the nanowires, and temperature. When the SiNW-FET at room temperature detects blood, λ_D_ is about 0.7 nm, and the Debye shielding effect at this time almost shields all the external electric field effects of the nanowires generated by the stacking of antigens. To effectively solve the Debye shielding effect, the principle is shown in Figure 4b. It is necessary to design a scheme to extend λ_D_ so that it exceeds the distance (d) between the antigen molecule and the nanowire, such as the purification antigen method [30,31]. In addition, some research groups have used some methods to shorten the d, such as the tailored antibody method [17] and the use of smaller sized aptamers [32] instead of antigens. Other teams have used high-frequency detection methods [33] or have modified a layer of biomolecule-permeable polymer in the nanowire to overcome Debye shielding [34].

In this experiment, our method to overcome Debye shielding was the dialysis membrane deionization method. Most of the salt ions in the detection solution were removed, thereby prolonging λ_D_ and overcoming the Debye shielding effect. We created a 10,000 dalton dialysis membrane into a microdialyzer. It can not only process red blood cells to obtain HbA1c but can also remove salt ions and overcome the Debye shielding effect.

The microdialyzer treats blood in two steps. The first obtains HbA1c by stepwise hemolysis. The principle is shown in Figure 4c−d. Figure 4e is a schematic diagram of normal blood, and HbA1c was located in red blood cells. In the detection, we needed to extract HbA1c. First, we needed to use 0.5 × PBS as the dialysate to reduce the blood salt ion concentration. Low concentrations of extracellular fluid caused swelling of the red blood cells and filtered outsome small blood molecules (Figure 4d). Then, we changed the dialysate to 0.25 × PBS to further reduce the concentration of the extracellular fluid until the red blood cells burst (Figure 4e), releasing HbA1c. Then, we obtained the HbA1c detection solution by simple anticoagulation tube centrifugation.

The second important role of the microdialyzer is dialysis desalination, and its desalting principle is shown in Figure 4f. The dialysis membrane enables salt ions and small blood molecules (such as creatinine, urea, uric acid, and glucose) to pass through the dialysis membrane along the concentration gradient [35]. However, various proteins (albumin, hemoglobin, and glycosylated hemoglobin) are retained in the blood. Due to the action of the dialyzer pressure sensor, the volume remains unchanged before and after hemodialysis. Therefore, the concentration of various protein molecules does not change. Figure 4g shows the ionic strength and Debye length of the solution during dialysis as a function of dialysis time. It can be seen that an ion concentration of 3 mM and a Debye length of about 6 nm could be achieved after 25–30 s of normal hemodialysis.

The device properties were tested and characterized after nanowire surface modification. We carried out a detection experiment, and the result of the detection experiment is represented by the output current Ids of the sensor. The quantitative detection experiment was divided into two parts, namely, standard detection and blood detection.

**Figure 5:**
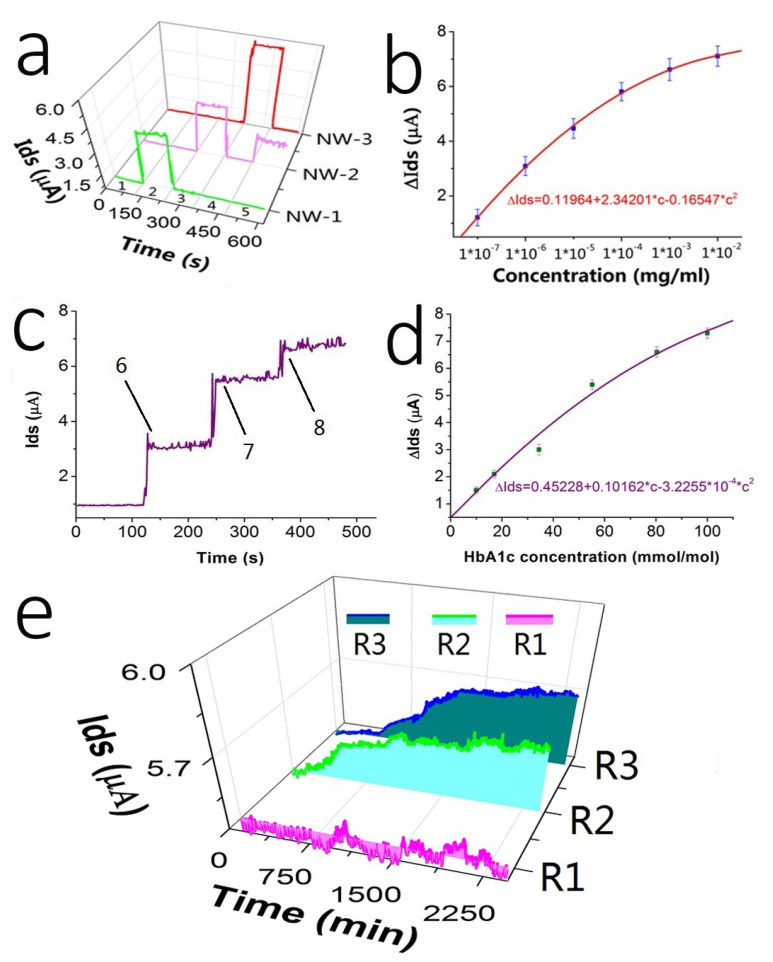
Experiment results of the detection application of SiNW-FET. ΔIds is represented by $\bar{x}$ ± SD, c represents concentration. (**a**) Specific detection result of the sensor, NW-1, NW-2, and NW-3 modified anti-GA, anti-HbA1c, and anti-BSA, respectively. They were detected in the following sequence: (1) 0.02 × PBS, (2) glycated albumin (GA) standard 5 ng/mL, (3) HbA1c standard 5 ng/mL, (4) bovine serum albumin (BSA) standard 5 ng/mL, and (5) HbA1c standard 0.5 ng/mL. (**b**) Results of current changes corresponding to different concentrations of the HbA1c standard. (**c**) Detection results of HbA1c in rabbit blood; the concentrations of HbA1c represented by 6, 7, and 8 were 34.43 mmol/mol, 55.19 mmol/mol, and 80.33 mmol/mol, respectively. (**d**) Results of current changes corresponding to different concentrations of HbA1c in rabbit blood. (**e**) Dynamic detection results of HbA1c.

The first part was the detection of standard products. We modified anti-GA, anti-HbA1c, and anti-BSA with three groups of nanowire device sensors, namely, NW-1, NW-2, and NW-3. In turn, five types of detection solutions were introduced into the three groups of sensors, and each detection solution was introduced for 120 s. The five detection solutions were (1) 0.02 × PBS, (2) GA standard 5 ng/mL, (3) HbA1c standard 5 ng/mL, (4) BSA standard 5 ng/mL, and (5) HbA1c standard 0.5 ng/mL. Figure 5a shows the current change curves for the three groups of sensors after the five groups of solutions were added sequentially. It can be seen that the sensor modified with the relevant antibody changed the current significantly only when detecting the relevant standard solution. This result can verify the specificity of the sensor. By comparing the current change curve of NW-2, it can be seen that the sensor presented different current changes when detecting different concentrations of standard solution. The higher the HbA1c concentration, the greater the current change. It shows that the sensor had a concentration–current-change correlation. We further detected HbA1c at different concentrations, as shown in Figure 5b, and we can see that the sensor current had changed according to different concentrations of the HbA1c standard. After the detection performance of the sensor was clarified through the detection experiment of the standard product, we continued to detect HbA1c in the blood of the experimental rabbit. Compared with standard products, blood testing requires microdialyzer treatment before detection. Microdialyzer treatment can not only release HbA1c in red blood cells but can also reduce the ionic strength of the test solution, thereby increasing the Debye length and overcoming the Debye shielding effect. Figure 5c shows the results of rabbit blood detection using the sensor modified with anti-HbA1c. The amount of HbA1c in the three tubes of rabbit blood was 34.43 mmol/mol, 55.19 mmol/mol, and 80.33 mmol/mol, respectively. Different current changes were generated with different concentrations, with Ids of 3.1 μA, 5.5 μA, and 6.7 μA, respectively. Next, we obtained the concentration–current-change correlation curve of Figure 5d for the detection of various HbA1c concentrations in rabbit blood. These results were similar to the HbA1c standard. It shows that the microdialyzer had the ability to obtain HbA1c and could effectively reduce the Debye shielding effect during detection.

Next, we attempted dynamic detection of HbA1c in experimental rabbits using our dialysis-SiNW-FET detection system. The detection experiment was divided into three groups. The experimental rabbit 1 was a normal experimental rabbit, and the normal feed solution (ter in die, 200 g/time) was injected from the gastric tube (set as R1). Experimental rabbit 2 was a diabetic model rabbit, and the diet and frequency were the same as those for rabbit 1 (but the feed ratio was 30% sucrose + 70% normal rabbit feed) (set as R2). Experimental rabbit 3 was a diabetic model rabbit, and the diet and frequency were the same as those for rabbit 1 (but the feed ratio was 40% sucrose + 60% normal rabbit feed) (set as R3). The detection time for the three groups was 40 h. The results are shown in Figure 5e. The currents of the R1 group of experimental rabbits were almost at the same level, indicating that the level of HbA1c was constant in the normal rabbits. The currents of the R2 and R3 groups showed a slow upward trend. This indicated that the concentration of HbA1c in the blood of the experimental rabbits slowly rose. This result indicates that SiNW-FET can realize dynamic detection of target molecules with changes in blood or serum concentrations. Unlike traditional quantitative detection, dynamic detection can characterize the continuous changes of a substance in an organism in a simpler, more efficient, and less damaging manner. This dynamic detection method enables earlier detection of the changes in HbA1c, which has great clinical application value.

## 4. Conclusions

SiNW-FET has good specificity and sensitivity in biological detection. When the microdialyzer is connected to the detection system, it can not only simplify the extraction process of HbA1c from red blood cells but can also reduce the salt ion strength in the detection solution. This overcomes the Debye shielding effect. More importantly, this experiment attempted the dynamic detection of biomolecules, which is the development direction of future detection technology and has great clinical value.

## Data Availability

Not applicable.
